# Digital Maturity as a Predictor of Quality and Safety Outcomes in US Hospitals: Cross-Sectional Observational Study

**DOI:** 10.2196/56316

**Published:** 2024-08-06

**Authors:** Anne Snowdon, Abdulkadir Hussein, Melissa Danforth, Alexandra Wright, Reid Oakes

**Affiliations:** 1 Department of Mathematics & Statistics University of Windsor Windsor, ON Canada; 2 Leapfrog Washington, DC United States; 3 Healthcare Information and Management Systems Society Chicago, IL United States

**Keywords:** digital health, readiness, cross sectional, observational, regression, digital maturity, association, associations, correlation, correlations, quality and safety, hospital performance, workforce, health outcomes, safety, service, services, healthcare system, healthcare systems, hospital, hospitals

## Abstract

**Background:**

This study demonstrates that digital maturity contributes to strengthened quality and safety performance outcomes in US hospitals. Advanced digital maturity is associated with more digitally enabled work environments with automated flow of data across information systems to enable clinicians and leaders to track quality and safety outcomes. This research illustrates that an advanced digitally enabled workforce is associated with strong safety leadership and culture and better patient health and safety outcomes.

**Objective:**

This study aimed to examine the relationship between digital maturity and quality and safety outcomes in US hospitals.

**Methods:**

The data sources were hospital safety letter grades as well as quality and safety scores on a continuous scale published by The Leapfrog Group. We used the digital maturity level (measured using the Electronic Medical Record Assessment Model [EMRAM]) of 1026 US hospitals. This was a cross-sectional, observational study. Logistic, linear, and Tweedie regression analyses were used to explore the relationships among The Leapfrog Group's Hospital Safety Grades, individual Leapfrog safety scores, and digital maturity levels classified as advanced or fully developed digital maturity (EMRAM levels 6 and 7) or underdeveloped maturity (EMRAM level 0). Digital maturity was a predictor while controlling for hospital characteristics including teaching status, urban or rural location, hospital size measured by number of beds, whether the hospital was a referral center, and type of hospital ownership as confounding variables. Hospitals were divided into the following 2 groups to compare safety and quality outcomes: hospitals that were digitally advanced and hospitals with underdeveloped digital maturity. Data from The Leapfrog Group's Hospital Safety Grades report published in spring 2019 were matched to the hospitals with completed EMRAM assessments in 2019. Hospital characteristics such as number of hospital beds were obtained from the CMS database.

**Results:**

The results revealed that the odds of achieving a higher Leapfrog Group Hospital Safety Grade was statistically significantly higher, by 3.25 times, for hospitals with advanced digital maturity (EMRAM maturity of 6 or 7; odds ratio 3.25, 95% CI 2.33-4.55).

**Conclusions:**

Hospitals with advanced digital maturity had statistically significantly reduced infection rates, reduced adverse events, and improved surgical safety outcomes. The study findings suggest a significant difference in quality and safety outcomes among hospitals with advanced digital maturity compared with hospitals with underdeveloped digital maturity.

## Introduction

Digital health is widely viewed as an opportunity to improve patient safety outcomes [1-5]. Digitally enabled health care has been proposed as an enabler for hospitals and health systems to advance and strengthen performance outcomes linked to the quadruple aim (ie, reduced costs, improved patient experience, improved workforce satisfaction, and better quality outcomes) [6-8].

Digital health technologies enable the collection of data to inform decisions to improve the quality and safety of care delivery, automate care processes to reduce errors, and improve the patient experience by engaging patients in their own health and care [1,2]. Although digital health tools and technologies have been proposed to enhance the quality and safety of health care, there is limited empirical evidence demonstrating the benefits of digital health technologies [1,2]. Specifically, research that has examined the benefits of digital health technologies on effectiveness and quality of care is skewed toward mental health services and behavioral therapies [2]. There are considerable gaps in the evidence of the impact of digital health transformation on cost, equitable care outcomes, and patient-centered care for hospitals or health systems [2].

The progress of digital transformation in health systems is often assessed using measures of digital maturity, which quantify the degree to which a health care organization has advanced digital capability, information infrastructure, and health data exchange, to provide digitally enabled health care services [9]. As health systems advance digital transformation efforts, the impact on organizational performance, workforce and workflows, and quality and safety outcomes for patients is required to document the return on investment to advance health system performance. However, evidence of the role of advanced digital maturity in strengthening these performance outcomes has not been well established empirically [10].

The progress of digital transformation in health systems has accelerated in recent years, fueled initially by incentives such as the Health Information Technology for Economic and Clinical Health (HITECH) act of 2009 [11,12]. The HITECH program provided financial incentives to US hospitals to adopt and implement electronic medical records (EMRs) with the goal of improving quality and safety of health care [11,12]. Over the life of the HITECH program, 98% of US hospitals participated in the program and received US $21.8 billion in federal incentives, and 60% of eligible office-based providers participated in the program and received US $16.2 billion in federal incentives. “Meaningful use” was defined in the HITECH program in terms of achieving required processes such as data collection requirements, adoption of secure email, and tracking the volume of messages, resulting in what some described as a “check the box” compliance program [12]. Although the adoption of EMR technologies has accelerated since the HITECH program, the actual use of advanced EMR functions such as physician order entry [13] and the use of data in clinical settings remain more limited [14].

Digital maturity model assessments have been used widely to document the adoption of EMR technologies and functions. One maturity model adopted widely in the United States is the Electronic Medical Record Assessment Model (EMRAM), developed by the Health Information Management Systems Society (HIMSS), a professional, not-for-profit organization with the mission of reforming the global health ecosystem through the power of information and technology [15]. The EMRAM model has been available in the United States for the last 15 years. Hospitals complete the online EMRAM survey of approximately 200 indicator statements that measure key dimensions of advanced EMR infrastructure and maturity including capacity for health information exchange, clinician adoption of digital tools, data privacy and security, and governance. Hospital teams receive a detailed assessment and report that indicate their level of digital maturity, strengths, and opportunities to advance their digital transformation strategy.

To date, there is clear evidence that the HITECH program accelerated EMR adoption across US health systems [13]. However, empirical research on performance outcomes such as quality, safety, and patient experience has been limited and may be influenced by the degree to which the full range of the functional features of EMR technologies has been adopted and scaled across provider organizations [16]. In addition, very few quality and safety programs in health organizations consider the use of digital technologies and associated risks of technologies used in health care, which further limits the evidence on how quality and safety outcomes are influenced by digital maturity [13]. More recently, significant attention to advances in digital transformation has emerged as an outcome of the COVID-19 pandemic, when a massive surge in demand for virtual care accelerated digitally enabled care services, which has continued to persist to varying degrees for specific patient populations [1]. Since the pandemic, quality and safety outcomes in US hospitals have reportedly declined, despite advances in digital transformation [17,18]. Since 2019, quality and safety outcomes for US hospitals have resulted in lower Leapfrog Group's Hospital Safety Grades (2023) [19]. Significant workforce shortages are common [20], which have further challenged hospitals to deliver quality and safe care. There is evidence that the adoption of EMR technologies is associated with added workload and burnout for the health workforce [21], which further underscores the importance of documenting the impact and value of advancing digital maturity on workforce, quality, and safety outcomes.

Numerous scholars and global agencies have examined quality and safety in hospitals and have documented gaps in care delivery whereby some patients receive health care services that are well below health and quality standards [19,22-26]. One in 10 patients seeking care in high-income countries are “adversely affected during care and treatment,” suggesting the need for significant improvements in quality of care across the globe [25]. Patient harm has become the 14th leading cause of global disease burden [27], which has resulted in significant harm for patients and contributed to substantial health system costs. Globally, 12% of national health expenditures are the result of managing the harmful outcomes resulting from unsafe care [28], which has fueled a renewed urgency for strengthening quality and safety outcomes among health care systems as a strategy to reduce health system costs. Effective interventions associated with improvements in quality and safety of care delivery include communication between clinicians and their patients [29,30], prevention of central line–associated blood infections (CLABSI) [31-33], use of hand hygiene practices to prevent infections [34,35], quality and safety training, established leadership priorities that focus on quality and safety [36], and improvements in clinical pathways [26,37,38]. More recently, there is evidence of the link between implementation of EMRs, improved patient outcomes [39], and cost benefits [40]. Notably, EMR-based interventions optimize digitally enabled care delivery (eg, digital communication between patients and clinicians; early detection of infections using predictive algorithms) and have all been proposed to improve quality and safety outcomes, particularly for hospital settings [40]. However, empirical evidence of the contribution of advances in digital transformation toward quality and safety outcomes is limited, which presents a challenge for health system leaders to make the case for investing in digital transformation efforts to advance quality and safety outcomes. The purpose of this research was to empirically examine quality and safety outcomes in US hospitals associated with advances in digital maturity.

## Methods

### Ethical Considerations

An ethics review board application was not submitted for this study as it did not meet the criteria for research ethics review, as this research does not involve human subjects or human biological materials (eg, human embryos, fetuses, fetal tissue, reproductive materials, and stem cells) [41]. This research used data available in the public domain and included only organizational level data associated with hospitals in the United States.

### Study Design

This research used a cross-sectional observational study design to examine the association between digital maturity and quality and safety outcomes in US hospitals. The analysis relied on organizational-level data that are publicly reported. The Leapfrog Group partnered with HIMSS on this research and provided The Leapfrog Group’s Hospital Safety Grades data set from the spring 2019 report (Table 1).

The Leapfrog Group is a national, not-for-profit organization in the United States that collects, analyzes, and publishes data on safety and quality outcomes in hospitals, intended to inform decisions to strengthen quality, improve safety, and reduce cost in US health care systems [42]. Leapfrog’s quality and safety measures include the Leapfrog Hospital survey; Leapfrog Hospital Safety Grade; Leapfrog Ambulatory Surgery Center Survey; and Leapfrog Value-Based Purchasing Program [42]. The Leapfrog Group administers 2 publicly reported hospital ratings programs annually. Publicly available data (eg, Centers for Medicare and Medicaid Services [CMS], the Leapfrog Hospital Survey, and secondary data sources such as the American Hospital Association’s Annual Survey and IT Supplement) are weighted and then combined to produce a composite score that is published as an A, B, C, D, or F letter grade for almost 3000 hospitals in the United States [43]. Austen et al [44] further described this methodology for arriving at a composite patient safety score.

A Leapfrog Group’s Hospital Safety Grade of “A” represents advanced patient safety outcomes achieved by acute care hospitals, and “F” represents safety outcomes that are lower than would be expected for an acute care hospital. The overall letter grades reported by Leapfrog are based on 28 individual quality and safety measures. In this study, we included 22 of the Leapfrog individual measures (Table 1). Variation in response rates was reported by Leapfrog for each safety measure resulting in different sample sizes for each Leapfrog measure included in this analysis (Table 1). The 2019 data set was selected for this analysis as it was the most complete data set compared with either 2020 or 2021, due to the impact of the COVID-19 pandemic on hospital participation.

**Table 1:** Leapfrog quality and safety variables included in the study.

Variable name (effective sample size)				Description		Analysis scale
Hospital safety grade (n=1026)				An overall letter grade based on a score calculated from 28 measures (see details in [6])		Categorical: A, B, C, D, or F
**Process/structural measures**						
	CPOE^a^ (n=911)		CPOE measures a hospital’s progress toward implementing a CPOE system to reduce medication ordering errors.		Fully meets the standard (Yes=1, No=0)	
	BCMA^b^ (n=902)		It measures (1) hospitals’ progress toward implanting BCMA throughout the hospital, (2) compliance with patient and medication scans during administration, (3) use of decision support, and (4) structures to monitor and reduce workarounds.		Fully meets the standard (Yes=1, No=0)	
	IPS^c^ (n=988)		It measures a hospital’s use of intensivists in ICUs^d^.		Fully meets the standard (Yes=1, No=0)	
**SP^e^**						
	SP1: Culture of Leadership Structures and Systems (n=615); SP2: Culture Measurement, Feedback & Intervention (n=615); SP4: Identification and Mitigation of Risks and Hazards (n=615); SP9: Nursing Workforce (n=615); SP19: Hand Hygiene (n=615)		These measure a hospital’s progress toward implementing NQF^f^-endorsed processes and protocols to reduce and prevent adverse events.		These variables are on continuous scales that are 0-120 for SP1 and SP2, 0-100 for SP4 and SP9, and 0-60 for SP19. Most of the hospitals achieved full scores. Therefore, our unit of measurement in this analysis is whether a hospital achieved a full score (Yes=1, No=0).	
**Outcomes**						
	**Hospital-acquired conditions**					
		FOR^g^ (n=1026); FAT^h^ (n=1026)	Three hospital-acquired condition measures are calculated by CMS^i^ through the DRA HAC^j^ Reporting Program: FOR after Surgery, Air Embolism, and FAT. Hospital-acquired condition rates are calculated by CMS based on Medicare Fee-for-Service claims.		Rate per 1000 inpatient CMS discharges on a scale of 0 to 1000	
	**Hospital-acquired infections**					
		CLABSI^k^ (n=759); CAUTI^l^ (n=882); SSI^m^: Colon (n=687); MRSA^n^ (n=641); C. Diff.^o^ (n=1026)	Hospitals were required to (1) join Leapfrog’s NHSN^p^ group, (2) provide an accurate NHSN ID in the Profile section of the Online Survey Tool, and (3) submit Section 7 Managing Serious Errors by November 30 for the purposes of calculating the Safety Grade.		These are measured as SIRs^q^ on a scale from 0 and unbounded above.	
**PSI^r^**						
	PSI 3: Pressure ulcer rate (n=1026); PSI 4: Death rate among surgical inpatients with serious treatable conditions (n=661); PSI6: Collapsed lung due medical treatment (n=1026); PSI 11: Postoperative respiratory failure rate (n=969); PSI 12: Perioperative PE/DVT^s^ rate (n=1026); PSI 14: Postoperative wound dehiscence rate (n=1026); PSI 15: Unrecognized abdominopelvic accidental puncture/laceration rate (n=1026)		PSI rates are calculated by CMS based on Medicare Fee-for-Service claims and publicly reported as a rate per 1000 inpatient discharges.		Rate per 1000 CMS eligible discharges on a scale of 0 to 1000	

^a^CPOE: computerized physician order entry.

^b^BCMA: bar code medication administration.

^c^IPS: intensive care unit physician staffing.

^d^ICUs: intensive care units.

^e^SP: safe practices.

^f^NQF: National Quality Forum.

^g^FOR: foreign object retained.

^h^FAT: falls and trauma.

^i^CMS: Centers for Medicare and Medicaid Services.

^j^DRA HAC: Deficit Reduction Act Hospital-Acquired Condition.

^k^CLABSI: central line–associated bloodstream infection.

^l^CAUTI: catheter-associated urinary tract infection.

^m^SSI: surgical site infection.

^n^MRSA: methicillin-resistant *Staphylococcus aureus*.

^o^C. Diff.: *Clostridioides difficile*.

^p^NHSN: National Healthcare Safety Network.

^q^SIR: standardized incidence rates.

^r^PSI: patient safety indicators.

^s^PE/DVT: pulmonary embolism/deep vein thrombosis.

The EMRAM maturity model data were made available by HIMSS for this analysis, as EMRAM has been widely available in the US market for the past 15 years. Hospitals complete an online assessment that has an embedded algorithm that generates a digital maturity level score on a 7-point scale of 0 through 7. Hospitals that score at a 6 or 7 digital maturity level are further assessed by an onsite HIMSS team to validate achievement of advanced digital maturity. Hospitals that do not meet the minimum EMRAM requirements for digital maturity are scored at level 0. For example, to achieve level 1 digital maturity, hospitals must have laboratory, imaging, pharmacy, and cardiology information systems fully integrated into the EMR to enable clinicians to access diagnostic reports and medication profiles, as well as resilience management plans to manage downtime of IT systems. For the purposes of this study, hospitals at level 0 on EMRAM were classified as having underdeveloped digital maturity. To be assessed at EMRAM level 0, data from laboratory, pharmacy, imaging, and interventional cardiology have not been integrated into the EMR, resulting in significant data silos. Underdeveloped digital maturity may indicate that the organization has not yet started their digital transformation journey or that the organization has not progressed its digital maturity beyond basic digital functionality. Hospitals assessed to have achieved stage 6 or 7 level of maturity were classified as achieving advanced digital maturity, which includes features such as ≥95% staff adoption and use of advanced EMR functions, including computerized physician order entry (CPOE), bar code medication administration (BCMA), integration of data from multiple internal and external sources and clinical settings, use of alerts to identify risk of errors, and automated digital tools to track progress of patient outcomes. Each of these advanced functions is designed to improve quality and safety, such as reduced errors related to physician orders or reduced errors during medication administration. Achievement of a level 7 EMRAM digital maturity indicates that the organization has reached advanced digital maturity. Requirements to achieve each subsequent level of digital maturity for the EMRAM model are described in Table 2. For this analysis, we focused only on hospitals at digital maturity levels of 0 (underdeveloped digital maturity) and level 6 or 7 (advanced digital maturity), as there were very few hospitals (eg, <20 hospitals) at maturity levels 1 through 5; these hospitals were excluded from the analysis due to a small sample size. The small number of hospitals scoring between levels 1 and 5 is viewed as the outcome of a number of factors, including lack of financial incentives to measure or advance digital maturity (eg, completion of HITECH program), limited financial and staff resources to complete assessments, competing priorities among leaders such as financial pressures, and workforce shortages, which have escalated since the pandemic. Hospitals that complete the EMRAM assessment and score level 0 may also be less inclined to repeat the assessment until such time as they have achieved significant progress in their digital maturity, whereby assessments of progress serve as motivation to profile their accomplishments when they reach level 6 or 7. The final study sample consisted of 1026 hospitals with complete Leapfrog Group’s Hospital Safety Grades (letter grades); complete EMRAM maturity level 0, 6, or 7; and complete CMS data describing hospital characteristics.

**Table 2:** Description of the Electronic Medical Record Assessment Model (EMRAM) level of digital maturity.

EMRAM level	Requirement to achieve stage
Level 0	The organization has not installed all the key ancillary department systems (eg, laboratory, pharmacy, cardiology, radiology).
Level 1	Laboratory, imaging, pharmacy, and cardiology systems produce patient-centric reports and results. Resilience management plans are in place.
Level 2	A CDR^a^ provides access to results and reports, governance and policy control, clinical decision support opportunities, training records, and IT security.
Level 3	Electronic clinical documentation is accessed remotely through the CDR. Role-based access controls are in place.
Level 4	Computerized practitioner order entry and electronic prescribing are available within an electronic medicines administration record. Clinical and information governance is well defined. Monitoring of clinical outcome and patient satisfaction targets occurs.
Level 5	Data are integrated from extremal sources. Changes in clinical parameters are continuously monitored by alerts and warnings. Telehealth and virtual care services are available. Intruder prevention systems manage unauthorized access. Technology supports bedside processes.
Level 6	Medical devices are integrated. Health information exchange supports data sharing. Service users submit self-reported outcomes data. Wearables and implants support remote monitoring and patient management of health and care. Online services improve access and health literacy.
Level 7	Data are integrated from multiple external sources. Service users receive alerts and reminders to support self-managed care and use automated tools to measure patient outcomes. Digital infrastructure tools enable dynamic patient engagement in managing personal health and care.

^a^CDR: clinical data repository.

### Statistical Analysis

Descriptive statistics were used to summarize the demographic features of hospitals included in this study. The number of beds was used to categorize hospital size as small (0-149 beds), medium (150-499 beds), and large (≥400 beds) hospitals. The outcome variables in this study differed in their scales of measurement and included binary variables, ordered categories, and continuous variables. The details of the analysis scales are reported in Table 1. Consequently, different types of regression modeling approaches were required to examine the associations between digital maturity levels (underdeveloped versus advanced) and The Leapfrog Group's Hospital Safety Grades, practices, and outcomes. The regression methodologies used in each case are described in the following paragraphs.

The association between EMRAM maturity and Leapfrog Safety (letter) grades, which is an ordinal categorical variable, was analyzed using a cumulative logit regression model, whereby the odds of achieving higher Leapfrog Safety Grades were estimated. EMRAM maturity levels were used as predictors while controlling for hospital characteristics (eg, bed size, ownership, rural or urban location, and teaching status).

The association between EMRAM maturity and the Process and Structural Measures of Leapfrog safety practices (CPOE, ICU, SP1, SP2, SP4, SP9, and SP19, described in Table 1) were analyzed using logistic regression analysis to model the odds of meeting the standard or achieving a full score with respect to EMRAM maturity levels and hospital characteristics.

The hospital-acquired infection (HAI) outcomes described in Table 1 had a substantial number of 0s, which makes ordinary regression models invalid. Therefore, the association between EMRAM maturity levels and HAI measures were analyzed using Tweedie regression, which is designed for outcomes that have nonnegative values with a large number of 0s [45]. The results of this analysis are presented as the multiplicative effects of digital maturity (ie, the predictor) on the average rate of the HAI outcome variables. For example, if the effect of EMRAM maturity on CLABSI is 1.2, it is to be interpreted as hospitals with underdeveloped maturity having 20% higher rates of CLABSI on average than digitally advanced (EMRAM 6, 7) hospitals.

Patient safety indicators (PSI3, 4, 6, 11, 12, 14, 15) and hospital-acquired conditions (“foreign object retention” and “falls and trauma,” described in Table 1), are reported by Leapfrog as the rate per 1000 CMS eligible discharges. These measures vary between 0 and 1000. The associations of these measures with EMRAM maturity and other hospital characteristics (eg, bed size, ownership, rural or urban location, teaching status) were analyzed using ordinary linear regression analysis. The results of this regression analysis are presented as the additive increases or decreases in average rates due to changes in the levels of the predictor variable (advanced versus underdeveloped maturity levels).

## Results

There were 1026 hospitals included in this study. Their characteristics are described in Table 3. The majority of hospitals in this study had not advanced their digital maturity, evidenced by EMRAM level 0 digital maturity.

The results revealed that, after controlling for hospital characteristics, the odds of achieving a higher Leapfrog Group's Hospital Safety Grade were statistically significantly higher, by 3.25 times, for hospitals with advanced digital maturity (odds ratio [OR] 3.25, 95% CI 2.33-4.55). When examining the role of hospital characteristics, nonteaching hospitals had better odds of achieving a higher Leapfrog Group’s Hospital Safety Grade, and governmental hospitals had lower odds of achieving a higher Leapfrog Group’s Hospital Safety Grade than both nonprofit and privately owned hospitals (Table 4). There was no statistical evidence that hospital size (eg, bed size) influenced Leapfrog Group’s Hospital Safety Grade outcomes.

The odds of achieving a full score on the culture of leadership structures and systems (SP1) was statistically significantly higher for hospitals with advanced digital maturity (OR 3.45, 95% CI 1.64-7.26). Similarly, such odds were 3.54-fold greater for digitally advanced hospitals with respect to nursing workforce (SP9; OR 3.54, 95% CI 1.53-8.18; Table 5). Although not statistically significant, the odds were also higher for hospitals with advanced digital maturity relative to identification and mitigation of risks and hazards (SP4) and hand hygiene practices (SP19; Table 5). Digital maturity was significantly associated with CPOE, intensive care unit physician staffing, and BCMA, whereby digitally mature hospitals had higher odds of meeting the standard for these 3 measures (Table 4). Advanced digital maturity was statistically significantly associated with a number of patient safety indicators, including the incidence of pressure ulcers (PSI3), episodes of collapsed lung (PSI6), and unrecognized abdominopelvic accidental puncture/laceration (PSI15; Tables 6 and 7). The majority of PSI outcomes for digitally mature hospitals indicated reduced rates of these adverse events.

CLABSI rates were an average 21% lower for hospitals with advanced digital maturity (Table 8), which was the only statistically significant association between digital maturity and HAIs. Large hospitals had significantly lower rates of CLABSI when compared with smaller hospitals, and nonprofit hospitals had significantly lower rates of CLABSI compared with either government or proprietary hospitals. Digitally advanced hospitals had, on average, lower rates of methicillin-resistant *staphylococcal aureus*, *Clostridioides difficile* infection, and surgical site infections, but the differences were not statistically significant.

**Table 3:** Demographic characteristics of hospitals.

Digital maturity level			Results, n (%)
Advanced (EMRAM^a^ 6,7)			162 (15.8)
Underdeveloped (EMRAM 0)			864 (84.2)
**Teaching status**			
	Teaching	475 (46.3)	
	Nonteaching	551 (53.7)	
**Rural vs urban status**			
	Rural	433 (42.2)	
	Urban	593 (57.8)	
**Ownership type**			
	Government	129 (12.6)	
	Nonprofit	666 (64.9)	
	Proprietary	231 (22.5)	
**Hospital size (number of beds)**			
	Small (1-149)	229 (22.3)	
	Medium (150-399)	180 (17.7)	
	Large (≥400)	615 (59.9)	
**Referral status**			
	Referral center	289 (28.2)	
	Nonreferral center	737 (71.8)	

^a^EMRAM: Electronic Medical Record Assessment Model.

**Table 4:** Association between digital maturity and Leapfrog Safety Grade, Structural, and Process Measures reported as odds ratios (ORs) and 95% CIs using a separate logistic regression model on each outcome (columns).

Independent variables (predictors)		Leapfrog Safety Grade, OR (95% CI)	CPOE^a^, OR (95% CI)	BCMA^b^, OR (95% CI)	IPS^c^, OR (95% CI)
Digital maturity (advanced vs underdeveloped)		3.25 (2.33-4.55)	2.29 (1.57-3.35)	1.51 (1.00-2.27)	1.75 (1.18-2.60)
Teaching (no vs yes)		1.38 (1.05-1.83)	0.98 (0.70-1.38)	0.96 (0.67-1.39)	0.78 (0.55-1.11)
Rural vs urban		1.2 (0.92-1.57)	0.78 (0.56-1.09)	0.62 (0.43-0.91)	0.77 (0.53-1.12)
Government vs nonprofit		0.67 (0.47-0.95)	0.89 (0.57-1.38)	1.31 (0.82-2.09)	1.05 (0.65-1.71)
Government vs proprietary		0.58 (0.39-0.86)	0.45 (0.27-0.73)	0.88 (0.52-1.47)	0.86 (0.50-1.48)
Nonprofit vs proprietary		0.86 (0.65-1.15)	0.51 (0.36-0.71)	0.67 (0.46-0.97)	0.81 (0.56-1.19)
**Hospital size**					
	0-149 beds vs 150-399 beds	0.98 (0.73-1.31)	0.62 (0.43-0.89)	0.73 (0.50-1.09)	0.22 (0.14-0.34)
	0-149 beds vs ≥400 beds	1.05 (0.71-1.56)	0.40 (0.25-0.66)	0.90 (0.53-1.53)	0.17 (0.10-0.29)
	150-399 beds vs ≥400 beds	1.08 (0.77-1.49)	0.65 (0.44-0.96)	1.22 (0.80-1.87)	0.78 (0.53-1.14)
Referral (no vs yes)		1.54 (1.14-2.09)	1.41 (0.97-2.05)	0.99 (0.66-1.49)	1.29 (0.86-1.93)

^a^CPOE: computerized physician order entry.

^b^BCMA: bar code medication administration.

^c^IPS: intensive care unit physician staffing.

**Table 5:** Association between digital maturity and safety practices (SP) reported as odds ratios (ORs) and 95% CIs using a separate logistic regression model on each outcome (columns).

Independent variables (predictors)		Culture of leadership (SP1), OR (95% CI)	Culture of measurement (SP2), OR (95% CI)	Risk mitigation (SP4), OR (95% CI)	Nursing workforce (SP9), OR (95% CI)	Hand hygiene (SP19), OR (95% CI)
Digital maturity (advanced vs underdeveloped)		3.45 (1.64-7.26)	1.46 (0.84-2.52)	0.76 (0.45-1.30)	3.54 (1.53-8.18)	1.34 (0.72-2.50)
Teaching (no vs yes)		0.61 (0.37-0.99)	0.89 (0.55-1.44)	0.77 (0.47-1.26)	0.53 (0.30-0.92)	0.82 (0.46-1.46)
Rural vs urban		0.52 (0.31-0.85)	1.05 (0.64-1.71)	0.96 (0.58-1.60)	0.81 (0.46-1.44)	0.94 (0.53-1.67)
Government vs nonprofit		0.41 (0.23-0.74)	0.59 (0.34-1.03)	0.49 (0.27-0.86)	1.57 (0.72-3.43)	0.52 (0.28-0.96)
Government vs proprietary		0.37 (0.20-0.70)	0.34 (0.18-0.65)	0.38 (0.20-0.74)	0.88 (0.38-2.05)	0.17 (0.07-0.38)
Nonprofit vs proprietary		0.91 (0.57-1.46)	0.58 (0.35-0.95)	0.79 (0.47-1.33)	0.56 (0.32-0.96)	0.32 (0.16-0.65)
**Hospital size**						
	0-149 beds vs 150-399 beds	0.94 (0.57-1.55)	0.65 (0.40-1.07)	0.71 (0.42-1.18)	0.94 (0.53-1.65)	0.64 (0.35-1.15)
	0-149 beds vs ≥400 beds	0.60 (0.28-1.31)	0.68 (0.34-1.35)	0.61 (0.29-1.27)	0.65 (0.27-1.57)	0.68 (0.30-1.56)
	150-399 beds vs ≥400 beds	0.65 (0.34-1.24)	1.04 (0.59-1.84)	0.86 (0.47-1.58)	0.69 (0.32-1.48)	1.07 (0.54-2.13)
Referral (no vs yes)		0.84 (0.48-1.47)	1.20 (0.71-2.04)	1.33 (0.77-2.29)	1.89 (1.03-3.46)	1.15 (0.62-2.14)

**Table 6:** Association between digital maturity and patient safety indicators (PSI) reported as mean change and 95% CIs using a separate linear regression model on each outcome (columns).

Independent variables (predictors)			Pressure ulcer rate (PSI3), mean change^a^ (95% CI)		Death rate serious conditions (PSI4), mean change^a^ (95% CI)		Collapsed lung (PSI6), mean change^a^ (95% CI)
Digital maturity (advanced vs underdeveloped)			0.06 (0.00 to 0.12)		1.87 (–1.39 to 5.13)		0.01 (0.00 to 0.02)
Teaching (yes vs no)			0.01 (–0.04 to 0.06)		–0.59 (–3.49 to 2.31)		0.01 (–0.00 to 0.01)
Urban (yes vs no)			0.00 (–0.05 to 0.05)		3.18 (–0.07 to 6.43)		0.00 (–0.00 to 0.01)
**Ownership (vs government owned)**							
	Proprietary ownership	–0.07 (–0.14 to 0.01)		–8.05 (–12.91 to –3.19)		–0.01 (–0.02 to 0.00)	
	Nonprofit ownership	–0.03 (–0.09 to 0.04)		–4.77 (–9.11 to –0.44)		–0.01 (–0.02 to –0.00)	
**Hospital size (vs small hospitals [0-149 beds])**							
	Large hospitals (≥400 beds)	0.09 (0.02 to 0.16)		–0.38 (–5.13 to 4.38)		–0.01 (–0.02 to 0.00)	
	Medium hospitals (150-399 beds)	0.03 (–0.02 to 0.09)		0.60 (–3.49 to 4.70)		–0.00 (–0.01 to 0.00)	
Referral (yes vs no)			0.00 (–0.06 to 0.06)		5.95 (2.68 to 9.23)		0.00 (–0.01 to 0.01)

^a^Average additive change in the mean of the measure with respect to a reference group.

**Table 7:** Association between digital maturity and patient safety indicators (PSI) reported as mean change and 95% CIs using a separate linear regression model on each outcome (columns).

Independent variables (predictors)		Postoperative respiratory failure (PSI11), mean change^a^ (95% CI)	Postoperative DVT/PE^b^ rate (PSI12), mean change^a^ (95% CI)	Postoperative wound dehiscence (PSI14), mean change^a^ (95% CI)	Accidental puncture/laceration (PSI15), mean change^a^ (95% CI)
Digital maturity (advanced vs underdeveloped)		0.46 (–0.02 to 0.94)	–0.05 (–0.21 to 0.11)	0.01 (–0.04 to 0.05)	0.07 (0.03 to 0.12)
Teaching (yes vs no)		0.25 (–0.16 to 0.67)	0.16 (0.02 to 0.29)	0.01 (–0.03 to 0.04)	0.01 (–0.03 to 0.05)
Urban (yes vs no)		0.14 (–0.26 to 0.55)	0.03 (–0.10 to 0.16)	–0.01 (–0.05 to 0.02)	–0.01 (–0.04 to 0.03)
**Ownership (vs government owned)**					
	Proprietary ownership	0.40 (–0.21 to 1.02)	–0.22 (–0.42 to –0.03)	–0.04 (–0.09 to 0.01)	–0.03 (–0.08 to 0.02)
	Nonprofit ownership vs government	–0.09 (–0.62 to 0.45)	–0.14 (–0.31 to 0.03)	–0.00 (–0.05 to 0.04)	–0.00 (–0.05 to 0.04)
**Hospital size (vs small hospitals [0-149 beds])**					
	Large hospitals (≥400 beds)	0.37 (–0.22 to 0.97)	0.19 (–0.01 to 0.38)	–0.04 (–0.09 to 0.01)	0.01 (–0.03 to 0.07)
	Medium-sized hospitals (150-399 beds)	0.38 (–0.05 to 0.81)	–0.11 (–0.25 to 0.03)	–0.00 (–0.04 to 0.04)	0.01 (–0.03 to 0.04)
Referral (yes vs no)		0.41 (–0.04 to 0.87)	–0.01 (–0.15 to 0.14)	–0.04 (–0.08 to –0.00)	–0.00 (–0.04 to 0.04)

^a^Average additive change in the mean of the measure with respect to a reference group.

^b^DVT/PE: deep vein thrombosis/pulmonary embolism.

**Table 8:** Association between digital maturity and hospital-associated infections and hospital-acquired conditions, reported as multiplicative mean change and 95% CIs using a separate Tweedie regression model on each outcome (columns).

Independent variables (predictors)		CLABSI^a^, mean change^b^ (95% CI)	MRSA^c^, mean change^b^ (95% CI)	CAUTI^d^, mean change^b^ (95% CI)	CDI^e^, mean change^b^ (95% CI)	SSI^f^, mean change^b^ (95% CI)	FOR^g^, mean change^b^ (95% CI)	FAT^h^, mean change^b^ (95% CI)
Digital maturity (advanced vs underdeveloped)		1.21 (1.02-1.42)	1.08 (0.91-1.29)	0.9 (0.78-1.05)	1.1 (0.99-1.22)	1.12 (0.94-1.33)	0.73 (0.39-1.35)	0.98 (0.83-1.16)
Teaching (yes vs no)		1.04 (0.9-1.19)	0.98 (0.84-1.14)	1.04 (0.91-1.18)	1.1 (1.01-1.2)	1.04 (0.89-1.22)	3.16 (1.51-6.62)	1.07 (0.93-1.24)
Urban vs rural		1.22 (1.04-1.43)	1.18 (0.99-1.4)	1.04 (0.91-1.2)	1.02 (0.94-1.11)	1.2 (1.01-1.43)	1.82 (0.92-3.56)	0.82 (0.71-0.95)
**Ownership (vs government owned)**								
	Proprietary vs government	0.81 (0.66-1.0)	0.83 (0.66-1.05)	0.78 (0.64-0.95)	1.03 (0.91-1.17)	0.65 (0.51-0.84)	0.33 (0.11-0.98)	1.09 (0.88-1.35)
	Nonprofit vs government	0.81 (0.67-0.97)	0.78 (0.64-0.96)	0.82 (0.69-0.97)	1.07 (0.96-1.2)	0.9 (0.73-1.1)	1.28 (0.53-3.06)	1.09 (0.91-1.32)
**Hospital size (vs small hospitals [0-149 beds])**								
	Large hospitals (≥400 beds)	1.24 (1.0-1.54)	1.1 (0.83-1.44)	1.06 (0.88-1.27)	0.98 (0.87-1.11)	1.33 (1.05-1.69)	27.11 (9.58-76.71)	1.30 (1.06-1.59)
	Medium hospitals (150-399 beds)	1.13 (0.95-1.35)	1.06 (0.83-1.35)	0.99 (0.85-1.14)	1.03 (0.94-1.12)	1.13 (0.92-1.38)	6.23 (2.44-16.12)	1.16 (0.99-1.35)
Referral (yes vs no)		1.12 (0.95-1.31)	1.14 (0.95-1.35)	1.08 (0.94-1.25)	0.99 (0.9-1.09)	1.21 (1.02-1.43)	1.51 (0.75-3.00)	0.89 (0.76-1.04)

^a^CLABSI: central line–associated bloodstream infection.

^b^Average multiplicative change in mean of the measure with respect to a reference group.

^c^MRSA: methicillin-resistant *Staphylococcus aureus*.

^d^CAUTI: catheter-associated urinary tract infection.

^e^CDI: *Clostridioides difficile* infection.

^f^SSI: surgical site infection.

^g^FOR: foreign object retained.

^h^FAT: falls and trauma.

## Discussion

This cross-sectional study included a large and diverse group of US hospitals (n=1026), whereby advanced digital maturity was a significant predictor of The Leapfrog Group's Hospital Safety Grades for hospitals of all sizes and types in this study. However, only 15.8% (162/1026) of these hospitals had achieved advanced digital maturity (assessed as EMRAM 6 or 7), with the majority of hospitals having underdeveloped digital maturity (scoring EMRAM 0). This finding is consistent with evidence of the impact of the HITECH policy in 2009, known as the “meaningful use” policy, which accelerated the adoption of EMR technologies in US hospitals but failed to stimulate adoption of the advanced EMR technology that is required to strengthen hospital performance, such as quality and safety [11,12]. The achievements of the 15.8% of hospitals with advanced digital maturity in this study offer insights into the key features of advanced digital maturity that are associated with stronger quality and safety outcomes. Hospitals validated as having advanced digital maturity are able to mobilize data from multiple external sources, have automated tracking of patient progress and health outcomes, and engage patients directly in accessing their health data to manage their health and care [15]. In contrast, the 84.2% of hospitals with underdeveloped digital maturity (EMRAM level 0) had not completed installation of all of the key ancillary information systems such as laboratory, pharmacy, cardiology, and imaging systems, which suggests that leaders and clinicians working in these hospital settings cannot readily track quality and safety outcomes or assess patient health status. When digital maturity is at a very basic level, data from these critical information systems cannot be readily accessed or mobilized, and significant limitations in the flow of quality and safety data result in lack of data-informed strategies and decision-making needed to advance quality, safety, and performance outcomes more generally.

A key question emerging from these findings is why so few hospitals have advanced their adoption of the advanced features of EMR technology. One potential explanation is the limited resources available to invest in advancing maturity without policy incentives such as the HITECH program to stimulate continued progress. Another possible explanation may be related to the profound impact of the pandemic on hospitals that experienced exceptional financial and workforce challenges from 2020 to 2023, whereby progress toward digital maturity likely halted or slowed down given the many competing demands during this challenging period. Finally, a third reason may be that hospital leaders in the United States could be hesitant to invest in digital maturity assessments as they progress their digital transformation effort until such time as they have made significant progress due to the concern that such results will be viewed as “poor or limited” progress among key stakeholders such as patients and funders. The US system is highly competitive, and leaders may be limited in their tolerance for maturity assessment outcomes of 0 to 4 that may have the potential to impact revenues and market reach.

In addition, hospitals with advanced digital maturity were significantly more likely to have a strong leadership safety culture, more advanced safety structures and systems, and stronger quality and safety outcomes that are sensitive to nursing workforce capacity (eg, risk assessments related to staffing, board accountability for safety-related staffing, budget resources that prioritize optimal staffing, and actionable strategies focused on performance improvement) [46]. Evidence of hospital strategies associated with advanced EMR adoption identify 3 key leadership and systems integration requirements [46]. Specifically, digitally mature hospitals have achieved advanced systems integration that offers the critical infrastructure capacity to support data-driven decision-making to advance quality and safety performance. A leadership culture that prioritizes safety may also be a critical driver of systems integration and advances in digital maturity that strengthen workforce capacity by integrating safe practices into workflows and establishing accountability structures to measure performance outcomes that are supported by a robust digital infrastructure for tracking and reporting safety outcomes.

The lack of integration of ancillary information systems evident in the majority of hospitals in this study may be a contributing factor to the negative impact of digital maturity on clinician workflows and clinician burnout, described in a number of studies [46,47]. Specifically, if lab, pharmacy, cardiology and imaging data do not flow automatically to an individual patient’s health record, then clinicians have few options but to search for these critical data points from multiple and disconnected software systems, which requires significant clinician time and attention. With the increased volume and complexity of patient demand, clinician workload is high, and the lack of system integration may further heighten the workload burden that has been reported relative to EMR technologies [47]. In addition, without the integration of data flows across the organization, there is very limited capacity for these hospitals to track organizational performance, track progress of patient outcomes, or proactively identify risks to enable interventions to mitigate risks to patients, in order to strengthen quality and safety outcomes.

The results of this study profile a number of key features of digitally advanced hospitals, including a strong leadership culture of quality and safety that leverages advanced digital maturity and EMR technologies as a strategy to achieve quality and safety performance, effective clinical workflows, workforce strategies that strengthen quality and safety (eg, staffing levels associated with accountability for safety outcomes), and data-rich work environments that automate data-driven decision-making and enable real-time tracking of performance outcomes to inform strategic decisions among hospital leaders.

Efforts to advance digital maturity in hospitals as a strategic priority have heightened since the pandemic, particularly given current workforce shortages and the decline in quality and safety performance [19]. The findings of this study support current evidence that advanced digital maturity strengthens quality and safety hospital performance outcomes [6-8], which previously had not been empirically well documented [10]. These findings may also offer policymakers and hospital leaders a strategic road map for advancing digital maturity as a strategy to improve quality and safety performance well into the future [17,18]. At a time when global workforce shortages are so challenging [20], these findings may also inform organizational strategies that advance quality of digital work environments that enable greater automation in flow of data to advance data-driven decision-making, reduce workload burden among clinicians, and support quality outcomes for patients [21]. Future policy levers may be effective in accelerating progress toward advanced digital maturity, focused on prioritizing quality and safety outcomes and reducing workforce burden, rather than focusing more narrowly on adoption of EMR technology [12].

New digital and sociotechnical trends in health care continue to emerge rapidly, including progress toward learning health systems [41], artificial intelligence technologies such as machine learning, and large language models such as generative artificial intelligence tools. All of these technologies rely heavily on a robust and mature digital infrastructure that is well integrated and able to capture, securely store, and flow data within and across clinical settings to inform health and care decisions. Yet, if the majority of US hospitals have not achieved digital maturity beyond level 0, then a significant number of hospitals in the United States may not have the digital maturity required to consider analytics strategies focused on adoption of these new technologies that hold so much potential for advancing quality care for patients. In addition, hospitals and health systems that have not advanced beyond level 0 maturity are likely to be profoundly limited in their capacity to advance learning health systems in which performance outcomes and quality of patient care are tracked and monitored to inform the design and development of new, digitally enabled models of care that ensure that every person in every health care setting has access to the highest quality of care possible.

In order to achieve the aspirational promises of digital transformation for US health systems, the findings from this study suggest that hospitals must accelerate progress toward advanced digital maturity to not only strengthen quality and safety outcomes for patients and quality work environments that support a robust and sustainable health workforce but also, perhaps most importantly, ensure that every hospital is well positioned to readily adopt and scale the many impressive digital tools and technologies such as artificial intelligence to ensure equitable and highly effective care delivery focused on sustaining and strengthening health and wellness for the US population.

There are a number of limitations in this study. The sampling frame of hospitals for this study was limited to organizations with a completed EMRAM assessment and Leapfrog survey in 2019, which may not represent all US hospitals. The second limitation is the lack of representation of hospitals at EMRAM maturity levels 1 through 5, which precluded examination of quality and safety advances at each level of EMRAM maturity. Very few hospitals assessed at EMRAM levels 1 through 5 are represented in the HIMSS US data set, which is a consistent pattern in other countries including Canada, Germany, and Australia. Hospitals typically assess their EMRAM maturity, initially at level 0, and then do not reassess again until they reach level 6, at which point they are validated with an onsite assessment by the HIMSS team.

The results of this study provide many opportunities for future research to document the role of digital maturity to better understand how it advances quality and safety. Specifically, future research may examine the role of data-driven decision-making, mobilizing data from multiple and diverse sources, automation of data flow, and digitally mature work environments that support clinicians to deliver quality care and enable active participation of patients in their care decisions. Future research that documents the complexity of care environments and the many features of digitally mature hospital care environments is needed to better understand how digital maturity advances quality and safe care that is equitable and offers every patient the opportunity to achieve their health and wellness goals. Future research may also examine the specific factors that have limited progress in advanced digital maturity for the majority of hospitals in this study. Examination of the key factors that preclude advancement of digital transformation may inform hospital leaders about strategies to overcome these barriers and challenges. The key feature of leadership expertise and strategy associated with learning health systems that create a culture of learning warrants further research with specific focus on how data-rich environments create the capacity for learning in health systems such as advances in quality of work environments, engagement of patients in their health and care, and effective management of the many competing demands to ensure every patient has the opportunity to access a strengthened health system performance that supports the health and wellness of every US citizen.

## Abbreviations

BCMA: bar code medication administration
CLABSI: central line–associated blood infection
CMS: Centers for Medicare and Medicaid Services
CPOE: computerized physician order entry
EMR: electronic medical record
EMRAM: Electronic Medical Record Assessment Model
HAI: hospital-acquired infection
HIMSS: Health Information Management Systems Society
HITECH: Health Information Technology for Economic and Clinical Health
PSI: patient safety indicator
